# Vimentin’s Journey from “Background Scaffold” to Multi-Scale Regulator of Neuronal Growth and Function: Historical, Conceptual and Epistemic Perspectives

**DOI:** 10.3390/ijms27114869

**Published:** 2026-05-28

**Authors:** Blen Amare Gebreselase, Alexander A. Minin

**Affiliations:** Institute of Protein Research, Russian Academy of Sciences, 119334 Moscow, Russia

**Keywords:** vimentin, intermediate filaments, neuronal cytoskeleton, neurite outgrowth, mitochondrial dynamics, neuronal development

## Abstract

Neurons achieve their highly polarized architecture by coordinating cytoskeletal systems across space and time, enabling axons to extend over remarkable distances and dendrites to elaborate complex arbours. Early neuroanatomists described intracellular “neurofibrils,” yet these structures remained poorly understood until electron microscopy resolved them into three distinct polymer systems: microtubules, actin filaments, and intermediate filaments. Although this framework clarified neuronal ultrastructure, it simultaneously established a conceptual hierarchy in which microtubules and actin were regarded as the principal drivers of neurite growth, while intermediate filaments were relegated to a passive, supportive role. Unlike prior reviews that document vimentin dynamics primarily from a cell-biological standpoint, this review integrates historical, conceptual, and epistemological perspectives to examine both how and why that hierarchy arose and how it has been dismantled. This review traces how that hierarchy arose and why it has been increasingly reconsidered in favour of intermediate filaments, focusing on vimentin as a case study. Evidence from live cell imaging, molecular manipulation, and genetic models shows that vimentin is dynamically regulated rather than static. Vimentin networks remodel continuously, exchange subunits with soluble pools, and move in coordination with microtubules. Most recently, sparse single-filament labelling combined with correlative volume electron microscopy has demonstrated that individual vimentin filaments remain motile even within dense perinuclear networks previously assumed to be static, a finding that fundamentally redefines what filament density implies about cytoskeletal organization. In neural and neural precursor cells, vimentin expression is developmentally regulated and is prominent during early differentiation stages associated with neurite initiation giving way to neurofilaments in mature neurons. Functional studies further link vimentin to neurite formation and extension, cytoskeletal coordination, organelle positioning, and cellular stress responses. Philosophical analysis reveals that these empirical advances were inseparable from shifts in imaging technology and conceptual framing, and that epistemic risks including model dependency and confirmation bias can be mitigated through methodological pluralism and explicit model disclosure. Taken together, these findings support a revised understanding of intermediate filaments as active, context-dependent contributors to neuronal development and plasticity, and illustrate the value of integrating biological evidence with historical and philosophical reflection.

## 1. Introduction

The extraordinary morphological diversity of neurons from short interneurons to metre-long motor axons demands a cytoskeletal system capable of both structural precision and dynamic adaptability. Early descriptions of intracellular “neurofibrils” using silver-staining methods helped establish neurons as discrete cellular units and supported the neuron doctrine, even before the molecular identities of these structures were known [1]. The subsequent advent of transmission electron microscopy was transformative: neuronal cytoskeletal elements became resolvable as distinct filament systems for the first time, revealing microtubules, intermediate filaments, and actin microfilament networks as separate and identifiable components [2,3].

Functional experiments in cultured neurons then made it possible to connect filament systems to growth behaviours. Disrupting actin polymerization or organization markedly alters growth cone structure and motility, while microtubule perturbation strongly affects axon elongation and stability [4,5]. These results entrenched a framing in which actin and microtubules were treated as the principal “active” engines of neurite growth. In contrast, intermediate filaments were long considered mechanically supportive elements whose contributions were indirect or redundant.

Over time, vimentin, one of the intermediate filament proteins, has become a useful case for revisiting that framing. Vimentin is developmentally regulated in the nervous system, appearing early in embryonic neural lineages and declining in expression as neurons mature [6,7]. More recently, live-cell imaging and genetic manipulations have demonstrated that vimentin networks are dynamic, are transported in microtubule-dependent ways, and can influence processes relevant to cell polarity and outgrowth [8,9,10]. Recent work has now extended these conclusions from ensemble behaviour to the level of individual filaments. Using sparse single-filament labelling and correlative volume electron microscopy, Renganathan et al. [11] demonstrated that individual vimentin filaments remain motile even within dense perinuclear networks previously assumed to be largely immobilized. These findings reveal that filament density does not imply static organization, and that vimentin dynamics persist at the single-filament level inside crowded cytoplasmic environments. This represents a critical shift from viewing vimentin dynamics as a bulk property to recognizing autonomous filament behaviour within intact cellular networks.

**Figure 1:**
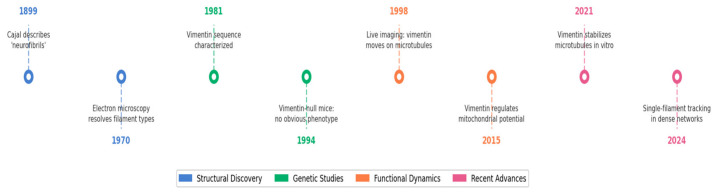
Timeline of key discoveries in vimentin and neuronal cytoskeleton research. Colour coding reflects conceptual era: structural discovery (blue), genetic studies (green), functional dynamics (orange), and recent ultrastructural advances (pink). The timeline illustrates the progressive shift from a static to a dynamic view of vimentin, driven by methodological innovation.

Building from these advances, the following sections trace how the neuronal cytoskeleton became an experimental object, how intermediate filaments and vimentin were positioned within it, and why current evidence supports a more integrated, multi-polymer view of neuronal morphogenesis. Table 1 and Figure 1 provide a chronological overview of advances in experimental approaches and the key discoveries that shaped our understanding of the role of vimentin biology.

## 2. Historical and Conceptual Development of the Neuronal Cytoskeleton

### 2.1. From Neurofibrils to Cytoskeletal Polymers

The intellectual origins of the neuronal cytoskeleton predate the term cytoskeleton itself. Late nineteenth century neuroanatomists, most prominently S. Cajal, described fibrillar networks within neurons using silver-staining approaches and interpreted them as organized internal structures consistent with neuronal polarity and discrete cellular boundaries [1]. Although the molecular identity of these neurofibrils was unknown, they functioned conceptually as an internal scaffold that rendered neuronal structure analysable and comparable across cell types.

A mid-twentieth-century instrumental shift brought transmission electron microscopy into neuroscience and transformed these qualitative fibrillar descriptions into distinguishable ultrastructural elements. Electron microscopy revealed filamentous components with characteristic diameters and morphologies within neuronal compartments, including microtubules and dense filament populations [2,3]. In developing and elongating axons, growth cones contain vesicles and membranous organelles, along with organized filament systems, whose spatial arrangement suggests specialized subregions for motility and structural support [3]. This period replaced a single neurofibrillary category with a pluralistic cytoskeletal architecture that could be systematically mapped to neuronal compartments.

Functional experiments reinforced this polymer-based framework. Early studies demonstrated that axon elongation depends both on the integrity of the axon shaft and on the continued activity of the motile tip, implicating distinct but coordinated roles for different filament systems during outgrowth [5]. Subsequent work using acute manipulation of actin organization in living growth cones showed that changes in actin structure rapidly reorganize growth cone morphology and influence microtubule behaviour within the same cellular domain, emphasizing functional coupling between cytoskeletal systems during guidance and advance [4]. Together, these findings established a tri polymer view of neuronal structure while also laying the groundwork for a hierarchy that positioned actin and microtubules as the primary dynamic agents of neurite growth.

### 2.2. The Rise of Intermediate Filaments and the Discovery of Vimentin

Intermediate filaments were distinguished from microtubules and actin filaments by their intermediate diameter and pronounced mechanical resilience. As biochemical fractionation and protein characterization matured, intermediate filaments were recognized as a diverse protein family with cell type and developmental specificity. Vimentin emerged as a major intermediate filament protein in mesenchymal and embryonic cells, and sequence analysis clarified its relationship to other intermediate filament classes such as desmin [28].

**Figure 2:**
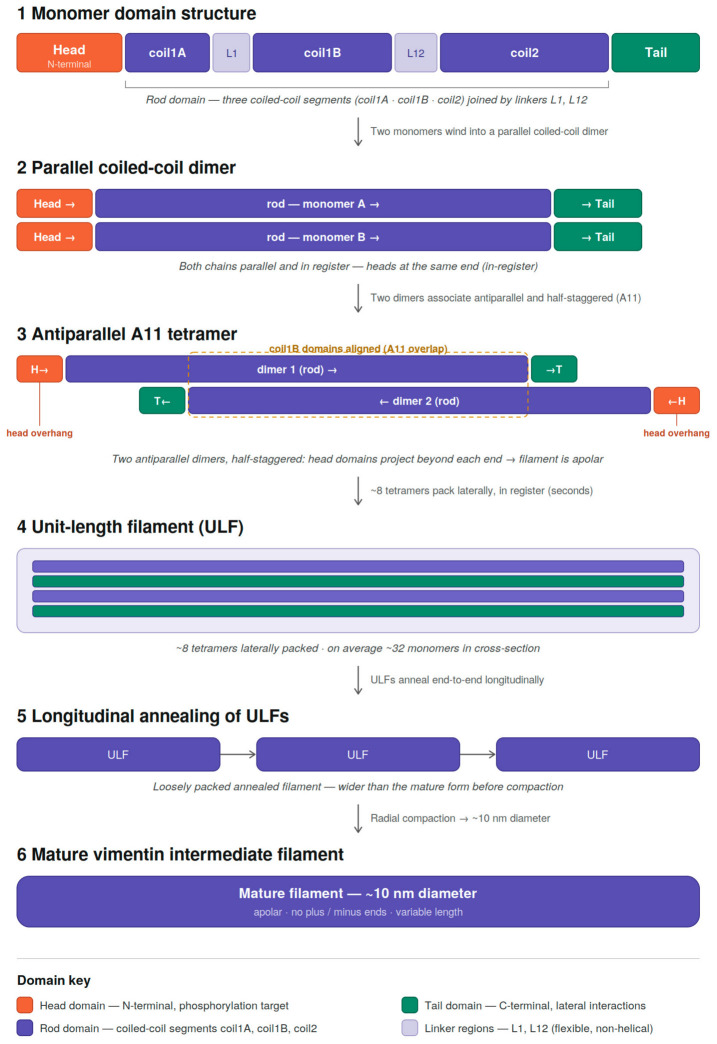
Schematic of vimentin domain organization and filament assembly pathway.

The domain organization of vimentin follows the canonical intermediate filament architecture, comprising a central alpha-helical rod domain flanked by a non-helical amino-terminal head domain and a carboxy-terminal tail domain. The rod domain mediates coiled-coil dimerization, which drives stepwise assembly from unit length filaments (ULFs) into mature filaments. Post-translational modifications, including phosphorylation of the head domain, regulate filament dynamics and soluble pool exchange (Figure 2).

A landmark demonstration that vimentin filaments assemble from soluble precursors established intermediate filaments as polymer systems with definable assembly intermediates, rather than as inert structural cables [14].

**Figure 3:**
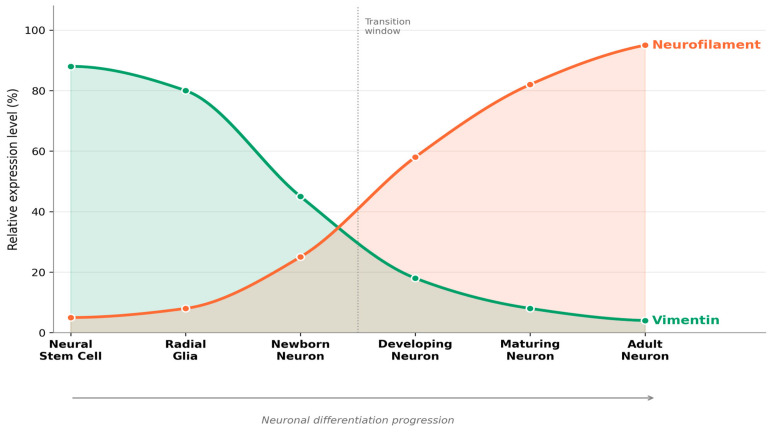
Cytoskeletal protein expression dynamics across neuronal differentiation. Smoothed area curves show relative expression (%) of vimentin (green) and neurofilament (orange) across six sequential stages. Vimentin predominates in neural stem cells and radial glia and declines as differentiation proceeds, whereas neurofilament rises and predominates in the mature neuron. The dashed vertical line marks the transition window during which vimentin and neurofilament levels cross.

Within the nervous system, immunohistochemical studies revealed that vimentin appears early during embryonic development and is present in immature neural cells (Figure 3). Vimentin was localized in embryonal glia and in early differentiating neurons both in vivo and in vitro, supporting the interpretation that vimentin marks early stages of neural development rather than mature neuronal identity [6]. Complementary in vivo studies charted the onset of intermediate filament expression during mouse embryogenesis and documented developmental patterns consistent with a transition in intermediate filament composition as neural cells differentiate [7].

Radial glia cells are particularly relevant in this context. These cells provide scaffolding for neuronal migration and also function as neural progenitors. Immunohistochemical analyses demonstrated an organized cytoarchitecture of radial glia in the embryonic mouse central nervous system, in which intermediate filament systems contribute to long cellular processes spanning cortical thickness [29]. Later work showed that dividing precursor cells in the embryonic ventricular zone exhibit morphological and molecular characteristics of radial glia, reinforcing the developmental setting in which vimentin is prominently expressed [30].

Together, these developmental observations suggested that vimentin could participate in early neurite formation and growth. However, because actin and microtubules were already closely associated with motility and intracellular transport, intermediate filaments were commonly interpreted as background structural support whose absence might be compensated by other cytoskeletal components. This interpretation persisted until advances in imaging rendered the dynamic behaviour of intermediate filaments directly observable.

Beyond the nervous system, vimentin is well established as a key marker and functional contributor to epithelial–mesenchymal transition (EMT), the process by which epithelial cells lose apical-basal polarity and acquire a migratory mesenchymal phenotype. Vimentin upregulation is a hallmark of the mesenchymal state, where it supports cytoskeletal remodelling, directional cell migration, and maintenance of cell polarity during tissue morphogenesis and tumour invasion [31]. Notably, the developmental dynamics of vimentin in neural precursor cells parallel those observed during EMT; in both contexts, vimentin is prominently expressed during periods of active cytoskeletal reorganization and is subsequently downregulated as cells adopt a more stable, differentiated identity [32]. This conceptual parallel reinforces the view that vimentin expression reflects a broadly conserved cellular programme of structural adaptability rather than a cell-type-specific scaffolding role.

### 2.3. Dynamic Intermediate Filaments and the Reconceptualization of the Cytoskeleton

The 1990s and early 2000s marked a major conceptual transition by making intermediate filament dynamics experimentally visible. Live-cell imaging of fluorescently labelled vimentin revealed continuous rearrangements in filament organization in interphase cells and showed that vimentin networks display motile behaviour rather than remaining fixed in place [10]. In parallel, studies identifying rapid, microtubule-dependent movement of vimentin-rich precursors demonstrated a transport mechanism linking intermediate filament organization to microtubule tracks and kinesin activity [9]. Together, these findings challenged the assumption that intermediate filaments serve only as static scaffolds by demonstrating that they undergo both subunit exchange and active transport.

Advances in imaging further refined this view. Live-cell structured illumination microscopy and photoconversion approaches enabled direct visualization of long vimentin filaments undergoing rapid bidirectional movement, showing that transport is a routine feature of intermediate filament behaviour rather than an exceptional state [8]. In vitro reconstitution and biophysical studies demonstrated that vimentin filaments stabilize microtubules against depolymerization and promote microtubule rescue, providing a physical basis for functional coupling between intermediate filaments and microtubules [24]. At the cell cortex, vimentin intermediate filaments and filamentous actin were shown to form interpenetrating networks, indicating that intermediate filaments can contribute directly to cytoskeletal organization across multiple spatial scales rather than being confined to deeper cytoplasmic regions [25].

Genetic models added an additional dimension to this reconceptualization. Mice lacking vimentin develop and reproduce without an obvious baseline phenotype, a result that initially suggested functional redundancy or compensation within the cytoskeletal system [17]. However, studies examining responses to injury and stress revealed more pronounced roles. Mice lacking both GFAP and vimentin display abnormal responses to central nervous system injury, indicating that intermediate filament systems can become critical under conditions where tissue mechanics and reactive programmes are engaged [18]. Together, dynamic imaging and context-dependent phenotyping reframed intermediate filaments as actively remodelling systems that interact with microtubules and actin, with functional importance that is most evident during development, reorganization, or stress.

## 3. Biological Functions of Vimentin in Neurite Growth, Mitochondrial Regulation, and Cellular Metabolism

### 3.1. Vimentin in Neurite Initiation and Growth

**Figure 4:**
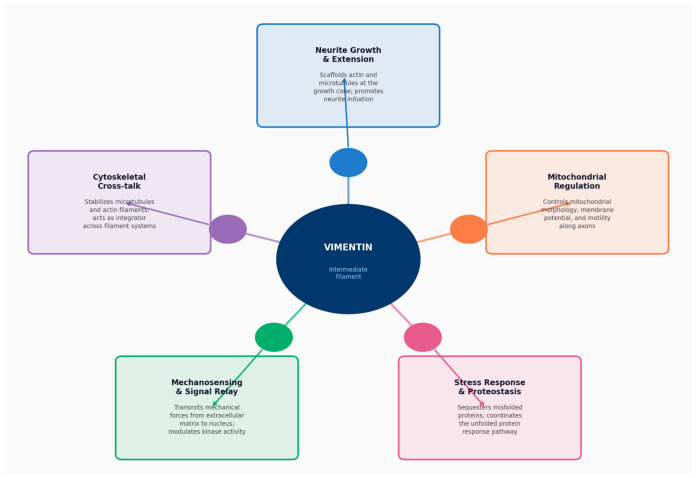
Multi-scale functional roles of vimentin in neural cells. Each satellite node represents a distinct biological function connected to the central vimentin network. All roles are mediated through vimentin’s dynamic interactions with microtubules and actin filaments. Solid line indicate regulatory influence; arrow direction indicates the direction of effect. The diagram highlights the hub-like nature of vimentin within neuronal cell biology.

A central question is whether vimentin contributes directly to neurite initiation and extension, or whether it functions primarily as a marker of developmental state. Early functional studies provided direct evidence for a role in neuritogenesis (Figure 4).

Intracellular delivery of anti-vimentin antibodies and reduction in vimentin expression using antisense oligonucleotides prevented neurite initiation in neuronal cells, while already-extended neurites were comparatively less affected, supporting a selective role during initiation rather than maintenance [16]. In cultured hippocampal neurons, antisense mediated reduction in vimentin similarly disrupted early neurite formation, consistent with a transient requirement during the earliest stages of process emergence [33]. In differentiated neuroblastoma cells, restoration of vimentin expression enhanced elongation of axonal neurites, suggesting that vimentin availability can limit growth under certain differentiation conditions [34]. More recent work has refined this view by demonstrating that vimentin plays a temporally restricted role during neuritogenesis. Parfenteva et al. showed that loss of vimentin selectively impairs early neurite initiation, while having comparatively modest effects on the maintenance of already extended processes [35]. These findings indicate that vimentin is not merely permissive for neurite growth but actively contributes to the cytoskeletal reorganization required for the establishment of neuronal polarity.

Mechanistic interpretations increasingly emphasize cytoskeletal integration rather than isolated filament effects. Vimentin dynamics depend on microtubules, and vimentin structures move along microtubule tracks through kinesin-linked mechanisms, providing a means by which intermediate filaments can influence polarized growth by coordinating local cytoskeletal organization [8,9]. While functional coordination between vimentin and microtubules has been well established, this coordination does not necessarily imply persistent or extensive physical co-alignment. Recent nanoscale analyses indicate that most vimentin filaments are spatially separated from microtubules, with direct interactions occurring only at low-frequency crossover sites. Importantly, individual vimentin filaments can transiently disengage from bundles, realign with microtubules, and undergo bidirectional motor-driven transport. These observations support a model in which sparse, transient interactions are sufficient to synchronize global cytoskeletal organization.

Direct physical interactions further support this view. Vimentin stabilizes microtubule dynamics in reconstituted systems, suggesting that it may support persistent microtubule growth or regrowth in regions undergoing structural remodelling [24]. At the cell periphery, vimentin participates in interpenetrating cortical networks with filamentous actin, offering an additional structural basis for influencing growth cone mechanics and protrusive behaviour at multiple length scales [25].

Post transcriptional regulation provides another link between vimentin and differentiation-associated neurite programmes. MicroRNA-124 promotes neurite outgrowth during neuronal differentiation and is associated with coordinated changes in cytoskeletal organization, positioning it as a regulator of neuritogenesis [36]. In motor neuron models, regulation of vimentin by microRNA-124 has been linked to changes in neuronal morphology, together with alterations in mitochondrial distribution, supporting the view that vimentin lies at an interface between differentiation programmes, neurite architecture, and organelle organization [37,38].

Disease-related human neuron studies further implicate vimentin-linked networks in neurite integrity.

In patient-derived midbrain neurons carrying SNCA duplication, combined transcriptomic and proteomic analyses identified dysregulation of vimentin-associated cytoskeletal pathways alongside neuritic deficits, consistent with altered neuronal morphology in a Parkinson’s disease genetic context [37,38]. In ALS motor neuron models, overexpression of miR-124, which directly targets vimentin, has been linked to marked changes in neuronal morphology and mitochondrial distribution, positioning vimentin within disease-relevant differentiation programmes and suggesting that its dysregulation contributes to motor neuron vulnerability [38,39]. More broadly, vimentin re-expression is a well-documented feature of the reactive glial response following traumatic brain injury and in chronic neurodegenerative conditions, where filament upregulation in astrocytes and other glial populations reflects a shift toward a reactive, injury-responsive cellular state [18,40,41]. Taken together, these findings indicate that vimentin dysregulation is not restricted to developmental contexts but extends across a range of neurological disease states, from familial Parkinson’s disease to ALS and acquired brain injury, strengthening the case for vimentin as a clinically relevant cytoskeletal component worthy of further investigation in neurological disease models.

Beyond these disease contexts, a related structural question concerns whether vimentin contributes to dendritic spine formation and remodelling processes that are disrupted across many of the same neurological conditions discussed above. Direct evidence for vimentin in mature dendritic spines is limited, consistent with the well-established developmental downregulation of vimentin as neurons mature. However, several indirect lines of evidence are suggestive. Vimentin is expressed in early dendritic processes during developmental stages when filopodial dynamics are most active. Furthermore, vimentin interacts with the actin cytoskeleton at the cell cortex [25], and actin is the primary cytoskeletal driver of spine morphogenesis. The mechanistic parallels between vimentin’s role in neurite initiation [16,33,35] and early filopodial protrusion suggest that vimentin may contribute to early dendritic elaboration even if it is not retained in mature spines. This represents a compelling direction for future investigation, particularly given the possibility of vimentin re-expression during synaptic plasticity or injury-induced remodelling.

### 3.2. Vimentin, Mitochondria, and the Spatial Organization of Metabolism

Mitochondria are central to ATP production and calcium buffering, and neurons depend on regulated mitochondrial distribution to meet local energy demands in distal cellular processes. In many cell types, mitochondria interact with cytoskeletal systems that control their transport and anchoring along microtubule tracks [42,43].

Primary studies demonstrate that vimentin intermediate filaments participate in this regulatory framework. Biochemical fractionation, immunoprecipitation, and fluorescence microscopy have demonstrated that vimentin associates with mitochondria, providing evidence for a physical connection between the vimentin filament network and these organelles [42]. Consistent with a role in spatial organization, cells lacking vimentin show increased mitochondrial motility and altered intracellular distribution, indicating that vimentin helps restrict mitochondrial movement and contributes to their positioning within the cytoplasm [22,44]. Beyond effects on motility, vimentin also influences mitochondrial morphology and network organization. Reduction in vimentin expression leads to mitochondrial fragmentation, swelling, and disruption of network integrity in cultured cells [19].

Vimentin-mediated coupling also affects mitochondrial membrane potential. Studies using vimentin-null fibroblasts and re-expression systems showed that the association of mitochondria with vimentin filaments increases membrane potential as measured by potential-sensitive dyes, whereas depletion of vimentin lowers membrane potential [21]. Regulation extends beyond static association. Rac1 signalling modulates interactions between mitochondria and vimentin and increases mitochondrial motility through a pathway involving PAK1, linking cytoskeletal signalling to mitochondrial dynamics and energetic state [22].

**Figure 5:**
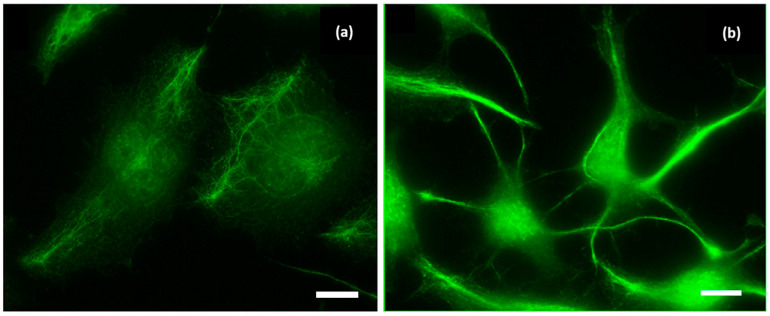
Cath.a (CAD) cells in serum-containing medium (**a**) and serum-free medium (**b**) after 24 h. Immunocytochemical staining with anti-Vim antibodies showing VIFs relocation in neurites during cell differentiation. Scale 20 µm.

In neuronal contexts, these relationships are directly relevant to morphogenesis. In CAD catecholaminergic neuronal cells (Figure 5), where serum withdrawal induces neurite formation [21], CRISPR Cas9-mediated knockout of vimentin reduced mitochondrial membrane potential, while reestablishment of wild-type vimentin restored this parameter. Reintroduction of vimentin network using its variants deficient in mitochondrial binding failed to rescue membrane potential. In the same system, neurites contained fewer mitochondria with high membrane potential when vimentin was absent, suggesting the hypothesis that vimentin contributes to coupling neurite growth states with local mitochondrial functional quality [25]. However, subsequent studies [44] revealed that vimentin’s influence on neurite outgrowth occurs independently of mitochondrial membrane potential. Collectively, these findings suggest that vimentin does more than simply bind to mitochondria and enhance their potential [19,20,45]; its role in promoting polarized neuronal growth appears to be more multifaceted.

### 3.3. Vimentin, Cellular Metabolism, and Stress Responses

The functional scope of vimentin extends beyond mitochondrial positioning into cellular stress handling and proteostasis processes that strongly influence metabolic resilience and lineage progression in neural stem and progenitor cells. In differentiating stem cell systems, vimentin has been shown to protect cells from stress: loss of vimentin increases sensitivity to stressors and impairs differentiation outcomes, including commitment to neuronal lineages [46].

In adult neural stem cells, a mechanistic connection between vimentin and proteostasis has been established through studies of aggresome biology during exit from quiescence. Following proteostasis challenge, neural stem cells form aggresomes, and vimentin organizes cage-like structures around these aggregates that can be asymmetrically segregated during mitosis. Vimentin is required for efficient recovery from these conditions; in its absence, neural stem cells show reduced capacity to exit quiescence and an earlier age-dependent decline in proliferation and neurogenesis, indicating a requirement for vimentin-mediated spatial organization of protein quality control machinery during activation [23]. Because exit from quiescence and early activation are energetically demanding transitions, these findings provide a route by which vimentin can influence metabolic robustness indirectly by supporting clearance and compartmentalization of damaged proteins during state changes.

Evidence from immune cells further supports the general principle that vimentin shapes metabolic outputs under challenge conditions. In neutrophils, loss of vimentin altered expression of genes linked to mitochondrial function and reactive oxygen species handling and was associated with increased reactive oxygen species and cytokine production that could be modified by targeting mitochondrial activity [47]. In macrophage-based models of atherosclerosis, vimentin deficiency increased oxidative stress markers and inflammatory responses, indicating that vimentin can limit oxidative stress in vivo in specific immune contexts [48]. Although immune and neural cells differ substantially, these findings support a shared mechanistic theme in which vimentin intersects with pathways that regulate how mitochondria-linked redox signals are translated into functional cellular responses.

### 3.4. Integrating Morphology, Organelles, and Metabolism

**Figure 6:**
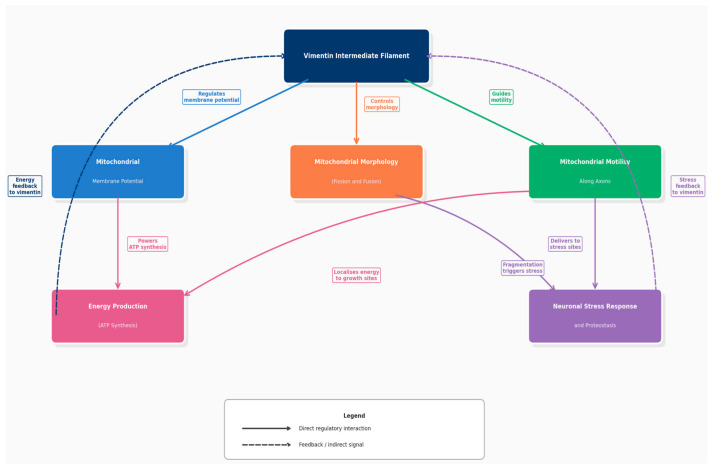
Vimentin–mitochondria regulatory circuit in neurons. All arrow labels describe the specific biological relationship between connected nodes. Vimentin intermediate filament exerts direct control over three mitochondrial properties (membrane potential, morphology, and motility along axons), each of which feeds downstream into energy production or the neuronal stress response. Two feedback loops (energy feedback and stress feedback) return information to vimentin, enabling adaptive regulation of the filament network.

Taken together, the primary literature supports a view of vimentin as a dynamic infrastructure that links neuronal morphology to organelle function and stress resilience, rather than as a passive cytoplasmic scaffold. Vimentin physically associates with mitochondria and shapes their morphology, motility, and intracellular organization (Figure 6) [19,20].

The observation that individual vimentin filaments can dynamically enter and exit loosely associated bundles provides a physical mechanism by which vimentin could locally regulate mitochondrial transport and anchoring without forming stable cytoskeletal tracks.

It also modulates mitochondrial energetic state through interactions that influence membrane potential, including neuronal cells undergoing neurite formation [21,49]. In parallel, vimentin organizes proteostasis machinery in neural stem cells by coordinating proteasome positioning at aggresomes and supporting efficient exit from quiescence, linking intermediate filaments to the capacity to manage proteotoxic stress during cell state transitions [23].

Conceptually, these converging findings support treating the cytoskeleton, including vimentin, as part of a cell’s metabolic geography. In this view, cytoskeletal systems help position mitochondria and quality control hubs where they are most needed during growth, differentiation, and remodelling. For neurons, whose physiology depends on spatially distributed demands at growth cones, synapses, and regeneration sites, this framework yields testable predictions. Altering vimentin organization should reshape not only filament architecture but also the spatial deployment of energetic capacity and stress-handling machinery during structural remodelling [20,23,49].

## 4. Experimental and Technological Approaches in Vimentin and Cytoskeleton Research

### 4.1. Classical Microscopy and Immunohistochemistry

The study of vimentin and the neuronal cytoskeleton has followed a technology-driven trajectory in which successive imaging modalities have reshaped both descriptive resolution and mechanistic inference. Early silver-staining approaches developed and refined by Ramón y Cajal revealed intracellular fibrillar structures that supported neuronal polarity and helped establish the neuron doctrine, even though the molecular composition of these fibrils remained unknown [1,50]. The later introduction of transmission electron microscopy enabled direct visualization of intracellular filaments, resolving these earlier fibrillar descriptions into distinct ultrastructural elements corresponding to microtubules, intermediate filaments, and actin-based structures [2].

Immunohistochemistry and immunofluorescence represented a further methodological advance by allowing molecularly specific labelling within intact tissue. Antibodies against vimentin, neurofilament subunits, and glial fibrillary acidic protein enabled precise mapping of intermediate filament expression across developmental stages and cell types. In the nervous system, these techniques revealed prominent vimentin expression in neural precursors, radial glia, and early differentiating neurons, with downregulation accompanying neuronal maturation [6,7,29]. Following injury, immunolabeling demonstrated robust re-expression of vimentin in reactive glial populations, reinforcing its association with cellular plasticity and remodelling states [18]. Beyond neuroscience, the same antibody-based approaches established vimentin as a canonical marker of mesenchymal and migratory states, strengthening conceptual links between vimentin expression, mechanical adaptability, and pathology [12].

### 4.2. Live-Cell Imaging of Intermediate Filament and Mitochondrial Dynamics

A decisive reconceptualization of intermediate filaments emerged with the adoption of live-cell fluorescence microscopy. The use of fluorescent protein fusions, such as GFP-vimentin, enabled direct observation of intermediate filament behaviour in living cells. Time-lapse imaging demonstrated that vimentin filaments undergo continuous remodelling, including elongation, shortening, and translocation, thus contradicting earlier assumptions of a static network [10].

Quantitative techniques such as fluorescence recovery after photobleaching and photoactivation further showed rapid subunit exchange between vimentin filaments and soluble pools, revealing dynamic turnover even within apparently stable filament networks. Subsequent advances in live-cell microscopy allowed visualization of long vimentin filaments undergoing rapid, bidirectional, and microtubule-dependent transport, demonstrating that filament redistribution is an active and regulated process [8,10].

Parallel developments in mitochondrial imaging enabled direct examination of cytoskeletal–organelle relationships. Fluorescent mitochondrial reporters and membrane-potential-sensitive dyes made it possible to track mitochondrial motility and energetic state in real time. Co-imaging experiments showed frequent spatial association between mitochondria and vimentin filaments and demonstrated that altering vimentin organization changes mitochondrial movement and membrane potential [19,20,21]. In neuronal and neuron-like cells undergoing neurite formation, live-cell imaging revealed that vimentin is required to maintain populations of high-potential mitochondria within growing processes, directly linking intermediate filament organization to local bioenergetics capacity [49].

### 4.3. Genetic Manipulation: From Knockouts to CRISPR-Based Editing

Genetic approaches provided an additional layer of insight by allowing sustained alteration of vimentin expression or structure. Early studies using vimentin-null mice reported relatively mild developmental phenotypes under baseline laboratory conditions, findings that initially suggested functional redundancy or compensation within the cytoskeletal system [17]. However, subsequent analyses revealed that the functional importance of vimentin becomes evident under demanding conditions such as CNS injury, oxidative challenge, or lineage transitions requiring extensive cytoskeletal remodelling [18,23].

The emergence of CRISPR–Cas9 genome editing has substantially expanded the scope of genetic analysis [51]. CRISPR-based deletion of vimentin in neuronal cell lines enables rapid generation of isogenic models and facilitates mechanistic interrogation of vimentin function at the cellular level [49]. More refined genome-editing approaches, including targeted mutagenesis of specific residues or interaction domains, provide a general framework for examining structure–function relationships of proteins in living cells [52]. CRISPR-based transcriptional repression and activation systems further permit graded modulation of endogenous gene expression without altering coding sequences, an approach established in pluripotent stem cells and their differentiated derivatives and applicable to dosage-sensitive regulators such as vimentin [53,54].

These genetic tools have also been incorporated into system-level analysis. In patient-derived neuronal models, vimentin loss has been used to reveal interacting pathways that shape neurite integrity and stress sensitivity, positioning vimentin as a node within broader genetic and functional networks rather than as an isolated structural element [39].

### 4.4. Transcriptomics and Systems-Level Views

High-throughput transcriptomic approaches introduced a systems-level perspective that reshaped how vimentin function is conceptualized. RNA sequencing enables unbiased, genome-wide assessment of expression changes associated with altered vimentin states in neural stem cells and differentiated neurons. In adult neural stem cells, transcriptomic analyses revealed coordinated changes in genes related to protein degradation, lysosomal function, mitochondrial metabolism, redox regulation, and cytoskeletal organization following vimentin loss, indicating broad reconfiguration of cellular programmes [23,46].

In disease-relevant human neuron models, combined transcriptomic and proteomic analyses identified dysregulation of cytoskeletal, mitochondrial, and proteostasis pathways associated with altered vimentin networks, linking intermediate filament organization to neurite deficits and cellular vulnerability [39]. These datasets shift explanatory focus from single mechanisms to network-level reorganization, highlighting vimentin as a regulator of coordinated cellular states rather than a single-purpose structural protein.

### 4.5. Cryo Electron Microscopy as an Epistemic Shift in Cytoskeletal Research

Cryo-electron microscopy and cryo-electron tomography represent a substantive change in standards of evidence by enabling direct visualization of cytoskeletal architecture in near native, vitrified cellular states. In contrast to earlier structural approaches that relied largely on purified proteins or chemically fixed samples, cryo-electron tomography allows intermediate filament organization to be examined within intact cells, preserving molecular context and spatial relationships. Notably, complementary advances in live-cell imaging now converge with cryo-electron microscopy to refine understanding of vimentin organization in intact cells. Sparse single-filament tracking combined with focused ion beam scanning electron microscopy demonstrates that vimentin bundles are loosely packed, structurally heterogeneous, and dynamically permeable. The convergence of live imaging and volume ultrastructural approaches underscores how methodological pluralism can overturn assumptions derived from ensemble-averaged observations [11].

Cryo-focused ion beam milling combined with cryo-electron tomography has resolved long-standing uncertainties about vimentin intermediate filament organization in situ. This work revealed a helical filament built from five protofibrils and showed that low-complexity head and tail domains contribute to distinct internal and lateral connections, providing a structural basis for mechanical behaviour and filament level variability [26]. In parallel, a later Perspective in Nature Cell Biology synthesized emerging work on how vimentin structure relates to mesenchymal cell mechanics and intracellular organization, framing cryo-electron microscopy as central to the current understanding of vimentin deformability [27]. Together, these developments illustrate a shift from inferred architectures toward directly observed molecular organization, while also highlighting practical constraints of cryo-based methods related to sample preparation, throughput, and access.

### 4.6. Methodological Limitations and Interpretive Challenges

**Figure 7:**
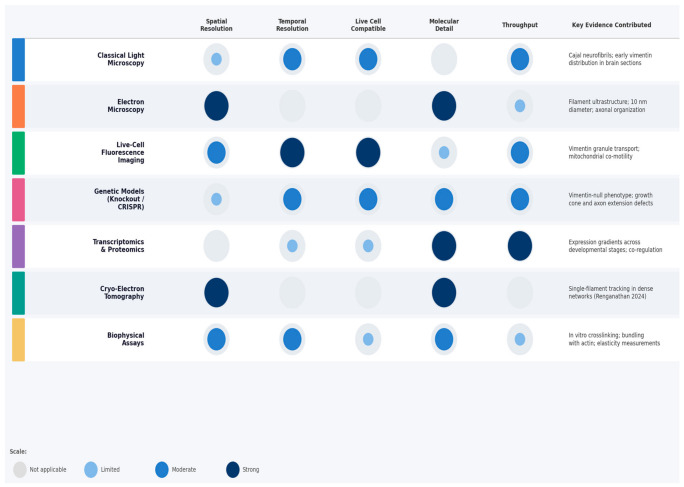
Methodological landscape in vimentin research: techniques, evidence, and constraints. Each row represents a technique; filled circles indicate performance across five dimensions (spatial resolution, temporal resolution, live-cell compatibility, molecular detail, and throughput). The final column summarizes the key empirical evidence contributed by each approach.

Despite substantial technical advances, each methodological approach introduces interpretive constraints (Figure 7). Live-cell imaging often relies on fluorescently tagged vimentin, which can influence filament organization if expression exceeds endogenous levels. Genetic deletion or transcriptional modulation of vimentin can induce compensatory changes in other cytoskeletal proteins or signalling pathways, complicating attribution of observed effects. Transcriptomic analyses typically reflect population averages and may obscure cell-to-cell variability, particularly in heterogeneous neural tissues.

Model systems introduce further complexity. Immortalized cell lines and primary cultures offer experimental accessibility but differ from intact brain tissue in architecture, extracellular matrix composition, metabolic environment, and activity patterns. Animal models provide greater physiological context but introduce species-specific differences and ethical constraints. Together, these considerations foreground a central epistemological issue: mechanistic insight into vimentin function is inseparable from the technologies and models through which it is observed.

## 5. Philosophical and Epistemological Considerations

### 5.1. Conceptual Frameworks and Research Priorities

The history of vimentin research illustrates how conceptual frameworks shape which biological questions are considered salient and which mechanisms are treated as explanatory. Early debates surrounding the neuron doctrine focused on whether the nervous system is composed of discrete cells or forms a continuous network, rather than on the internal molecular organization of neurons themselves [50]. Within this context, intracellular fibrillar structures served primarily as anatomical evidence supporting cellular individuality rather than as mechanistic objects of study.

Once cytoskeletal polymers became identifiable through microscopy and biochemical methods, explanatory attention increasingly shifted to microtubules and actin filaments. Their rapid dynamics and direct involvement in growth cone motility, intracellular transport, and neuronal polarity positioned them as the dominant explanatory entities for axon growth and synaptic remodelling, as reflected in later syntheses of the field [55,56]. In contrast, intermediate filaments, including vimentin, were long interpreted as providing primarily passive structural reinforcement and were therefore assigned a secondary role in neuronal explanation, a view that has been explicitly described and critically reassessed in more recent reviews [57].

This conceptual division influenced research priorities by directing experimental effort, funding, and theoretical modelling toward actin and microtubule systems. Intermediate filaments attracted less-sustained investigation until unexpected observations revealed that they undergo continuous remodelling, interact with other cytoskeletal systems, and participate in processes relevant to neuronal growth and metabolism. From a philosophical perspective, this pattern reflects the theory-laden character of observation: what researchers notice, measure, and interpret is shaped by prior theoretical commitments about which kinds of entities are likely to matter [58,59,60]. In the case of vimentin, shifts in available imaging and labelling techniques, together with new empirical findings, prompted a revision of those commitments, allowing intermediate filaments to be reconsidered as dynamic contributors to cellular organization. Researchers can apply this lesson directly by explicitly stating their working model of vimentin when designing experiments, ensuring that conceptual assumptions remain visible and open to revision. The assumption that dense filament networks are intrinsically static illustrates how methodological constraints shape biological interpretation. Earlier ensemble-labelling approaches were incapable of resolving single-filament behaviour within crowded regions, reinforcing a conceptual hierarchy that privileged microtubules and actin as the primary dynamic elements of the cytoskeleton. The ability to visualize autonomous filament motion within dense vimentin networks now demonstrates how advances in experimental access can destabilize entrenched explanatory frameworks and reassign causal significance within cytoskeletal systems.

### 5.2. Molecular Reductionism and Its Limits

Research on vimentin also illustrates both the strengths and limitations of molecular reductionism in neuroscience. On the one hand, explaining neuronal phenomena in terms of molecular components has yielded productive results. Identifying vimentin as a contributor to neurite initiation, mitochondrial organization, and stress tolerance enables experimentally precise questions and links cellular behaviour to defined molecular interactions. This approach aligns with strong forms of reductionism that aim to explain neural and cognitive phenomena directly through molecular and cellular mechanisms [61].

At the same time, the vimentin literature reveals limitations of a simple reductionist narrative. Vimentin-related effects are strongly dependent on developmental stage, cellular context, and environmental conditions. In many systems, removal of vimentin produces modest phenotypes under baseline conditions, while functional consequences become evident only during differentiation, injury, or metabolic challenge. Moreover, vimentin acts through distributed interactions involving other cytoskeletal polymers, organelles, and signalling pathways, rather than through a single linear causal chain.

Philosophical work on mechanistic explanation provides a framework that accommodates these features. Mechanistic accounts explain phenomena by describing how entities and activities at multiple levels are organized to produce a given outcome [62,63]. Within such accounts, vimentin is understood as one component within mechanisms that generate neurite growth or metabolic adaptation, where its contribution depends on spatial arrangement, temporal coordination, and interaction with other components. Mechanistic explanations also allow for reciprocal relationships across levels, in which molecular organization is constrained by cell architecture and developmental history, and higher-level changes can influence filament organization and gene expression [62,64]. This perspective captures why vimentin cannot be fully understood in isolation, yet still remains a legitimate target of molecular explanation. This suggests that mechanistic studies of vimentin should routinely probe its function alongside partner proteins and under physiologically relevant conditions rather than in isolation.

### 5.3. Experimental Models and Epistemic Risk

A further epistemological issue concerns the experimental models through which vimentin function is studied and the risks associated with generalization. Much of the mechanistic evidence for vimentin roles comes from experimentally tractable systems such as fibroblasts, immortalized neural progenitor lines, primary neurons grown in vitro, or rodent neural stem cells [23,46]. These models enable controlled manipulation and detailed measurement but differ substantially from the structural, metabolic, and activity-dependent conditions of intact human brain tissue.

Animal models provide access to in vivo organization and developmental processes, yet they introduce additional sources of uncertainty, including species-specific differences in cytoskeletal expression patterns, lifespan, and disease progression. Emerging human-based systems, such as induced pluripotent stem-cell-derived neurons and brain organoids, reduce some translational gaps but raise questions about maturation state, network organization, and long-term stability.

Philosophers of biology emphasize that model choice is not neutral. Each model embodies assumptions about which features of a phenomenon are essential and which can be idealized or ignored [62,64]. In vimentin research, prioritizing early developmental or injury-related models may emphasize roles in neurite initiation while underrepresenting functions related to long term maintenance, ageing, or disease progression. Recognizing these epistemic risks supports a pluralistic strategy that integrates evidence across multiple models and levels of analysis and treats extrapolation to human cognition and pathology with appropriate caution [63,65]. Practically, combining complementary model systems, such as primary neuron cultures, with iPSC-derived neurons can reduce these risks while broadening the biological conclusions that can be drawn.

### 5.4. Technological Mediation and the Visibility of Mechanisms

Technological change does not merely provide finer descriptions of an already known biological reality. It actively shapes which mechanisms become observable and, as a result, which explanations are considered viable. A clear example in neuroscience is the discovery of the periodic actin-spectrin lattice in axons, a structural organization that remained undetected until the development of super-resolution microscopy. Once visualized, this lattice required revisions to prevailing models of axonal mechanics and membrane organization, demonstrating how changes in visibility can reorganize explanatory priorities [66,67]. Subsequent computational studies have further explored the mechanical consequences of this architecture [68].

A similar pattern characterizes research on vimentin. Dynamic relationships between vimentin filaments and mitochondria or protein aggregates became experimentally accessible only after the adoption of live cell imaging, organelle-targeted fluorescent probes, and reporters of stress and autophagy pathways. These approaches revealed coordinated movements, changes in mitochondrial membrane potential, and spatial coupling between vimentin networks and proteostasis machinery that were not evident in fixed-cell or biochemical assays [19,21,23,46].

Transcriptomics and other omics technologies have introduced a further shift in explanatory practice. Instead of inferring mechanisms solely from targeted molecular measurements, researchers increasingly rely on patterns of co-regulation and differential expression across thousands of genes. In vimentin-related studies, such approaches have uncovered coordinated changes in pathways associated with protein degradation, mitochondrial metabolism, redox regulation, and immune signalling [23,39,46]. While these datasets expand the range of candidate mechanisms, they also raise interpretive challenges, as correlations alone do not establish causal organization. From a philosophical perspective, this reinforces the need to integrate data-driven discovery with focused experimental tests that specify how proposed components contribute to a mechanism.

### 5.5. Speculative Frameworks and Cytoskeletal Complexity

At the margins of mainstream neuroscience, some theorists have proposed that cytoskeletal proteins may participate directly in higher-order cognitive processes. Proposals centred on microtubules, and more tentatively on intermediate filaments, have suggested roles in information processing or consciousness, although such ideas remain controversial and lack broad empirical support [69].

Regardless of their ultimate plausibility, the existence of these proposals reflects a broader shift in how cytoskeletal proteins are conceptualized. Structures once regarded as inert supports are now widely recognized as dynamic organizers of cell shape, signalling, and metabolism. From a philosophy of science perspective, speculative cytoskeletal theories can be understood as attempts to extend mechanistic explanation into new explanatory domains. Their scientific value depends on whether they generate testable predictions and whether they can be integrated with established biological evidence.

In the case of vimentin, the strongest empirical support currently lies at the level of neurite dynamics, organelle organization, and cellular stress responses. Nevertheless, vimentin’s extensive interactions with signalling pathways, transcriptional programmes, and metabolic processes suggest that its functional reach extends beyond simple mechanical support, even if it does not warrant claims about direct involvement in cognition itself [57,70].

## 6. Conclusions

The historical trajectory of research on the neuronal cytoskeleton, and on vimentin in particular, illustrates how scientific understanding emerges through the interplay of experimental tools, conceptual frameworks, and philosophical commitments. Early descriptions of an indistinct neurofibrillary network gave way to a polymer-based view of neuronal structure through advances in microscopy and immunohistochemistry, ultimately establishing microtubules, actin filaments, and intermediate filaments as distinct but interacting components [50].

Within this framework, vimentin moved from the periphery of neuronal biology to a position of functional significance. Once treated primarily as a marker of immature or reactive states, vimentin is now recognized as contributing to neurite initiation, mitochondrial organization, metabolic regulation, and cellular stress responses in neural and neural precursor contexts [19,21,23,33,49]. These advances depended on methodological innovations, including live cell imaging, genome editing, transcriptomics, and, most recently, cryo-electron tomography, which made dynamic behaviour and structural heterogeneity directly observable [8,26]. Philosophical analysis highlights that these empirical developments are inseparable from shifts in explanatory stance. Simple distinctions between structural and regulatory proteins, or between dynamic and static cytoskeletal elements, have given way to multi-level mechanistic accounts in which components are understood through their organized interactions across scales [62,63]. At the same time, choices of experimental model and technology introduce epistemic risks by foregrounding some aspects of vimentin function while obscuring others.

These epistemic risks can be actively mitigated through several complementary strategies. Methodological pluralism is essential, as conclusions about vimentin function are most robust when supported by convergent evidence from multiple independent approaches rather than from any single technique alone. Explicit disclosure of the experimental model used and its known limitations reduces the risk of over-generalizing findings from cell lines or rodent models to human biology. Replication across cell types and species guards against model-specific findings becoming entrenched as universal principles. Finally, cross-disciplinary dialogue between cell biologists, biophysicists, and philosophers of science can surface hidden assumptions that silently guide experimental design and interpretation. Adopting these practices would make epistemic risk visible and therefore manageable.

Taken together, vimentin serves as a revealing example of how a single cytoskeletal protein can participate in the coordination of morphology, organelle behaviour, and metabolic state. It also illustrates the value of integrating historical awareness, empirical rigour, and philosophical reflection when evaluating molecular explanations in complex biological systems. Continued progress in this area will likely depend on methodological pluralism, careful interpretation of high-dimensional data, and sustained attention to the conceptual assumptions that guide research on neuronal form and function.

## Figures and Tables

**Table 1:** Chronological overview of advances in imaging technologies and the key discoveries that shaped our understanding of vimentin intermediate filaments biology.

Timeline	Key Discovery/Conceptual Shift	Experimental Approach/Technology	Significance/Epistemic Impact	Representative Citations
**Late 1800s–1910s**	Neurofibrils described as intracellular fibrous structures; support neuron doctrine	Silver staining (Cajal’s method), light microscopy	First visualization of neuronal internal structure, though molecular identity unknown	[1]
**1950s–1970s**	Cytoskeletal polymers resolved: microtubules, microfilaments, and intermediate filaments distinguished	Transmission electron microscopy (TEM), heavy metal staining	Transformed “neurofibrils” into distinct structural categories; enabled polymer-specific hypotheses	[2,3]
**1978**	Vimentin identified as a distinct intermediate filament protein	Protein biochemistry, SDS-PAGE, peptide mapping	Established vimentin as molecularly definable entity, not just morphological feature	[12]
**1981**	Vimentin sequence determined; relationship to desmin established	Protein sequencing, cDNA cloning	Enabled molecular probes (antibodies, oligonucleotides) for specific detection	[13]
**1982–1984**	Vimentin expression mapped in developing nervous system: radial glia, neural precursors	Immunohistochemistry, immunofluorescence	Linked vimentin to developmental state, not just cell type; suggested functional role in neurogenesis	[6,7]
**1983**	Vimentin assembles from soluble precursors	Pulse-chase biochemistry, in vitro assembly assays	Demonstrated filaments are dynamic polymers, not static cables	[14]
**1991**	Vimentin moves along microtubules; transport is kinesin-dependent	Video microscopy, microtubule co-sedimentation, kinesin inhibitors	First evidence of active transport; linked intermediate filaments to motor proteins	[15]
**1993**	Vimentin required for neurite initiation in neuroblastoma	Anti-vimentin antibody microinjection, antisense oligonucleotides	Functional evidence in living neurons; suggested active role in morphogenesis	[16]
**1994**	Vimentin-null mice generated—viable with no overt phenotype	Homologous recombination in embryonic stem cells	Initially suggested redundancy; later revealed context-dependent functions under stress	[17]
**1998**	Live vimentin dynamics visualized: filaments move, elongate, shorten	GFP-vimentin fusion, time-lapse fluorescence microscopy	Decisive reconceptualization: intermediate filaments are dynamic, not static	[9,10]
**1999**	Vimentin/GFAP double-null mice show impaired CNS injury response	Immunohistochemistry, injury models (spinal cord lesion)	Revealed functional importance under stress; context-dependent roles	[18]
**2008**	Vimentin binds mitochondria; influences mitochondrial morphology	Immunoprecipitation, fluorescence microscopy, siRNA knockdown	Extended vimentin’s functional reach to organelle regulation	[19]
**2011**	Vimentin modulates mitochondrial motility	Live imaging of mitochondrial reporters, vimentin-null fibroblasts	Established vimentin as spatial organizer of organelle distribution	[20]
**2015**	Vimentin regulates mitochondrial membrane potential	Potential-sensitive dyes (TMRM, JC-1), vimentin-null cells, re-expression	Linked intermediate filaments to bioenergetics, not just structure	[21,22]
**2015**	Rapid bidirectional transport of long vimentin filaments	Live-cell structured illumination microscopy (SIM), photoconversion	Revealed transport as routine, not exceptional; single-filament resolution	[8]
**2020**	Vimentin organizes aggresomes in neural stem cells; required for quiescence exit	CRISPR-Cas9 knockout, proteostasis reporters, live imaging	Connected vimentin to protein quality control and stem cell activation	[23]
**2021**	Vimentin stabilizes microtubules directly; promotes rescue from depolymerization	In vitro reconstitution, TIRF microscopy, purified proteins	Molecular mechanism for vimentin-microtubule coordination	[24]
**2022**	Vimentin forms interpenetrating networks with actin at cell cortex	Super-resolution microscopy, structured illumination, genetic manipulation	Revised understanding of cortical cytoskeleton; vimentin reaches periphery	[25]
**2024–2026**	Cryo-ET structure of vimentin in situ: helical filament, five-protofibril architecture	Cryo-FIB milling, cryo-electron tomography	First near-native structure; revealed low-complexity domain organization	[26,27]
**2025–2026**	Single vimentin filaments tracked within dense networks; autonomous motion demonstrated	Sparse single-filament labelling, FIB-SEM volume microscopy, particle tracking	Overturned assumption that dense networks are static; single-filament agency	[11,28]

## Data Availability

No new data were created or analyzed in this study.
